# Redox Homeostasis and Regulation in Pluripotent Stem Cells: Uniqueness or Versatility?

**DOI:** 10.3390/ijms222010946

**Published:** 2021-10-11

**Authors:** Julia S. Ivanova, Olga G. Lyublinskaya

**Affiliations:** Institute of Cytology of the Russian Academy of Sciences, Tikhoretskii pr. 4, 194064 St. Petersburg, Russia; o.lyublinskaya@mail.ru

**Keywords:** pluripotent stem cells, ROS, redox homeostasis, redox signaling, redox metabolism, proliferation, differentiation, somatic reprogramming

## Abstract

Pluripotent stem cells (PSCs) hold great potential both in studies on developmental biology and clinical practice. Mitochondrial metabolism that encompasses pathways that generate ATP and produce ROS significantly differs between PSCs and somatic cells. Correspondingly, for quite a long time it was believed that the redox homeostasis in PSCs is also highly specific due to the hypoxic niche of their origin—within the pre-implantation blastocyst. However, recent research showed that redox parameters of cultivated PSCs have much in common with that of their differentiated progeny cells. Moreover, it has been proven that, similar to somatic cells, maintaining the physiological ROS level is critical for the regulation of PSC identity, proliferation, differentiation, and de-differentiation. In this review, we aimed to summarize the studies of redox metabolism and signaling in PSCs to compare the redox profiles of pluripotent and differentiated somatic cells. We collected evidence that PSCs possess metabolic plasticity and are able to adapt to both hypoxia and normoxia, that pluripotency is not strictly associated with anaerobic conditions, and that cellular redox homeostasis is similar in PSCs and many other somatic cells under in vitro conditions that may be explained by the high conservatism of the redox regulation system.

## 1. Introduction

Oxygen is a critical factor for the existence of all aerobic organisms. Redox reactions, inherent in aerobic metabolism, are among the most abundant chemical interactions in living cells. Low molecular weight oxidants called reactive oxygen species (ROS) play a key role in these reactions. ROS are ions and neutral molecules, often in the form of free radicals, that are more reactive than molecular oxygen. In total, more than a dozen different substances belong to the ROS family—superoxide anion radical, hydrogen peroxide (H_2_O_2_), hydroxyl radical, singlet oxygen, etc. (a detailed list can be found in the review [1]). At the onset of redox biology, ROS were considered to be only the toxic by-product of cellular respiration, but later it became clear that ROS play an important role in metabolic and signaling processes that regulate cell proliferation, differentiation, motility, and apoptosis [1,2,3,4]. The sources of ROS are usually subdivided into mitochondrial and non-mitochondrial. The main producers of mitochondrial ROS are several enzymatic complexes of the mitochondrial electron transport chain (ETC) [5,6], whereas the main, but not single, non-mitochondrial ROS generator is a family of NADPH oxidases (NOXs) [7]—in total, more than 40 enzymes are known to produce ROS [1]. The main mediator of redox signaling is H_2_O_2_, which is enzymatically produced in different cellular compartments—either directly in reactions involving molecular oxygen or by dismutation of superoxide anion radical, also formed from O_2_ [8]. In parallel with the intracellular generation of ROS, their rapid elimination constantly occurs, and enzymatic systems responsible for that compose the antioxidant defense system of the cell. ROS are removed by highly productive enzymes such as superoxide dismutases, peroxiredoxins, glutathione peroxidase, and catalase [9]. These enzymes provide precise control of intracellular ROS, keeping them at a very low level—in particular, about 10^−9^ M in the case of H_2_O_2_ and 10^−11^ M in the case of superoxide anion radical [1].

The situation when intracellular ROS level exceeds the physiological norm is called oxidative stress, or more specifically, ‘oxidative distress’ [10]. Under conditions of distress, high oxidizing ability of ROS causes extensive damage to cell macromolecules—proteins, lipids, and nucleic acids [11]. In this regard, in the early studies of cellular redox homeostasis, the main attention was paid to the negative impact of oxidative distress induced by external factors or redox metabolism disorders. To cope with the oxidative stress conditions, it was proposed to use pharmacologic antioxidants, defined as substances that “delay, prevent or remove oxidative damage to a target molecule” [12]. Later it was found that elevated ROS production accompanies and worsens the course of many human diseases, such as cardiovascular, neurodegenerative, oncological, and others. From that moment on, antioxidants were actively tested for use in therapy; however, surprisingly, clinical trials did not meet the expectations, often having no effect or leading to a worse prognosis [13,14]. Failures in using antioxidants for clinical practice, together with the accumulated fundamental knowledge on cellular redox systems, have brought to the forefront studies of molecular mechanisms that ensure the maintenance of normal redox homeostasis as well as research on the regulatory role of ROS in metabolic and signaling processes. In contrast to the term ‘oxidative distress’, for the description of the physiological ROS level modulation that performs signaling or regulatory functions the term ‘oxidative eustress’ was introduced [10,15].

The shift of the paradigm that took place in redox biology at the turn of the century proceeded in parallel with the development of stem cell research. The derivation of pluripotent embryonic stem cells (ESCs) from mouse (mESCs) [16,17] and then from human (hESCs) [18] at the very end of the 20th century became a turning point in stem cell and developmental biology, as well as in regenerative medicine. ESCs give rise to the formation of all tissues and organs of an organism. The proliferative and differentiating potential of these cells ensures the development of the embryo; therefore, the ability to study the properties of these cells and induce their differentiation into various cell types of three germ layers in vitro is of particular importance for both fundamental and practical aspects of biomedicine. The use of differentiated descendants of ESCs for the treatment of various human pathologies initially held a great promise [19], but the question of histocompatibility remained open. Thus, further development of a technology for obtaining induced pluripotent stem cells (iPSCs) from adult cells by ectopic expression of only four transcription factors [20,21] has once again spurred interest in the study of pluripotency.

Pluripotent stem cells (PSCs), both ESCs and iPSCs, have a number of unique characteristics that distinguish them from somatic cells: a clonal growth capacity, shortened proliferation cycle, high ability for DNA damage repair, and inability to activate the programs of cellular senescence, due to, among the other things, telomerase activity inherent in these cells [18,22]. PSCs also have specific metabolic features corresponding to their initial localization within the blastocyst in the hypoxic conditions of the uterine cavity. While the mitochondrial electron transport chain (ETC) is the main energy source in somatic cells, PSCs rely to a greater extent on anaerobic glycolysis even when cultured at atmospheric oxygen levels [23,24,25]. Their mitochondria possess morphological and functional differences in comparison to the mitochondrial network of differentiated cells [24]. Since mitochondrial enzymes of ETC are one of the main sources of intracellular ROS, highlighting the peculiarities of PSC energy metabolism led to the concept that the redox homeostasis in PSCs significantly differs from that of their differentiated progeny cells and that PSCs can limit intracellular ROS production to minimize ROS-induced oxidative damage. However, recent studies have shown that PSCs have not only phenotypic but also metabolic plasticity and, therefore, are able to adjust their redox metabolism to the conditions of their microenvironment [26,27]. These cells a priori have the capacity to exist not only in hypoxic conditions but also in normoxia (21% O_2_) [26] and even hyperoxia (>21% O_2_) [28]. Recently published data show that under conditions in vitro, redox parameters of PSCs have much in common with the redox characteristics of differentiated cells [29,30]. Moreover, in PSCs, similarly to somatic cells, maintaining the physiological ROS level turned out to be critical for the regulation of their identity, proliferation, differentiation, and de-differentiation [31]. In this review, we aimed to summarize the studies investigating the redox processes and redox parameters of PSCs and tried to answer the questions: (1) what is in common between the redox profiles of PSCs and somatic cells; (2) which characteristics of redox homeostasis and signaling can be considered as distinctive features of PSCs, and which ones are highly conservative and thus similar in pluripotent cells and their differentiated descendants?

## 2. Redox Homeostasis in Pluripotent Stem Cells

### 2.1. Quantification of the ROS Level in PSCs

The main characteristic of cell redox homeostasis is an intracellular ROS level, which is usually determined by assessing the ability of a whole cell (or its individual compartments) to oxidize redox probes. Fluorescent compounds (dyes or genetically encoded biosensors), prone to change their fluorescence upon oxidation, are generally used as such probes. Some of them (e.g., many biosensors [32]) are specifically oxidized by individual ROS compounds, but for the most part, redox probes are capable of being oxidized by the variety of intracellular oxidants and are commonly used to assess the overall ROS level in cells [33,34].

**Figure 1:**
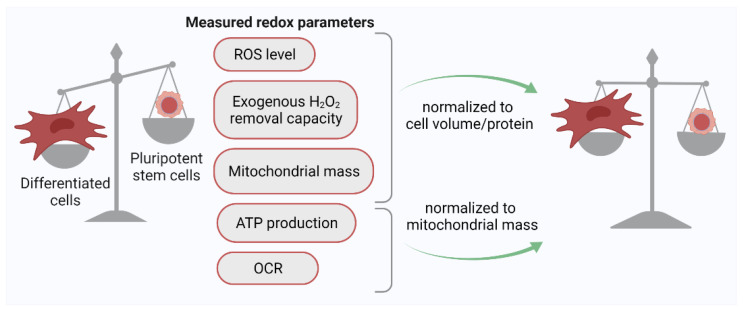
Normalization of key redox parameter values to cell protein/volume or mitochondrial mass leads to the leveling of these parameters between PSCs and their differentiated progeny cells. ROS, reactive oxygen species; OCR, oxygen consumption rate.

The first studies aimed to assess the level of ROS in pluripotent cells were carried out using the most common among all redox-sensitive dyes, 2′,7′-dichlorodihydrofluorescein diacetate (H2DCFDA). H2DCFDA is originally fluorescently inactive but starts to emit intense fluorescence upon oxidation. This probe can react with various intracellular oxidants; however, it mostly is oxidized by H_2_O_2_ [34]. In 2004, it was found that the level of ROS in cultured mESCs appeared to be several times lower than that in their spontaneously differentiated progeny cells, as well as in embryonic mouse fibroblasts and mouse fibroblast line 3T3 cells [28]. Since then, numerous studies have shown that both murine and human PSCs (ESCs, iPSCs) have very weak ability to oxidize H2DCFDA in comparison to their differentiated counterparts [35,36,37]. Experiments using other ROS-sensitive dyes (peroxide-sensitive DHR 123 [35,38], superoxide-sensitive DHE [37], mitochondrial superoxide-sensitive MitoSOX [35,38]) showed similar results. At first, these observations were explained by the peculiarities of the redox metabolism in PSCs. It is known that these cells originate from the preimplantation blastocyst, which is surrounded by an intrauterine fluid with an oxygen content of about 4% [39,40]. Considering the hypoxic conditions in which PSCs exist in the body, a hypothesis about the low ability of pluripotent cells to generate ROS was formulated (discussed in [41]). According to this hypothesis, low generation of ROS in PSCs allows preventing oxidative damage to cell proteins, lipids, and DNA. However, later on, more detailed studies of intracellular redox environment in hESCs carried out in comparison with their fibroblast-like descendants, as well as differentiated human cells of different nature and origin (lymphocytes, fibroblasts, mesenchymal stem cells, HeLa cells), have shown that the differences in the cell capacities to oxidize redox-sensitive probes depend strongly on the cell sizes [30]. For the adequate comparison of the redox status of different cells using the H2DCFDA probe, it was proposed to use normalized parameters: the ratio of the H2DCFDA signal to the cell volume (biophysical normalization) or cell protein (biochemical normalization). The use of this approach showed that the ROS level evaluated on a per volume or per protein basis does not differ significantly between PSCs and differentiated human cells (Figure 1). Thus, it eventually appeared that PSCs and their differentiated progeny cells are equally committed to generating ROS, at least under normoxic (21% O_2_) culture conditions.

### 2.2. ROS Production in PSCs

#### 2.2.1. Mitochondrial Activity in PSCs

Mitochondrial ROS generation is closely related to cell bioenergetics. Cellular energy is produced in the processes of multistage oxidation (dehydrogenation) of various substrates (sugars, proteins, lipids, amino acids) formed during the catabolism of nutrients consumed by the body. Some stages of this oxidation process occur in the cell cytoplasm, and others—inside mitochondria, but all of them are coupled to the production of ATP. Mitochondrial ATP synthesis is accompanied by generation of ROS. The ATP and ROS outputs in cellular mitochondria depend thus on the activity of mitochondria [42].

The first stage of glucose breakdown (glycolysis), which takes place in the cytoplasm, does not require oxygen. During glycolysis, ATP is synthesized by substrate phosphorylation (i.e., by attaching to ADP a phosphate group after it is cleaved from phosphate-containing compounds). In the course of glycolysis, pyruvate is formed, which can be further converted into lactate under anaerobic conditions. Alternatively, pyruvate can be transported into mitochondria where, after decarboxylation, it associates with coenzyme A. The product of this reaction, Acetyl-CoA, participates in the next aerobic, intra-mitochondrial stage of glucose decomposition in the tricarboxylic acid (TCA) cycle, during which the acetyl residues (CH_3_CO-) are oxidized to carbon dioxide (CO_2_). Along with glucose degradation products, the TCA cycle can be fueled by breakdown products of other substrates (lipids, amino acids) [42]. Compounds generated in the TCA cycle, in turn, support the work of the electron transport chain, a series of protein complexes found on the inner mitochondrial membrane. ETC produces ATP through oxidative phosphorylation (OXPHOS) reactions, in which ATP synthase catalyzes the addition of inorganic phosphate to ADP. It is worth noting that besides its role in oxidative catabolism of carbohydrates and fatty acids, the TCA cycle also provides precursors for many biosynthetic pathways, including precursors for amino acid and nucleotide synthesis [25]. The production of mitochondrial ROS (mitoROS) is interlinked with bioenergetic metabolism via the OXPHOS. Electron leak from ETC leads to the generation of a highly reactive metabolite of molecular oxygen, superoxide anion (O_2_^−^·), formed by one-electron reduction of O_2_. O_2_^−^·, in turn, is rapidly dismutated to H_2_O_2_ by two dismutases—Cu/Zn-superoxide dismutase (SOD1) in mitochondrial intermembrane space and Mn-superoxide dismutase (SOD2) in mitochondrial matrix. In the reactions of H_2_O_2_ with transition metal ions, the hydroxyl radical (OH·) is formed. In spite of the fact that free radical ROS are highly reactive, their lifetime is extremely short, while H_2_O_2_ is a much more stable and long-lived molecule.

First studies devoted to the bioenergetics of PSCs showed that these cells in comparison with their differentiated progeny cells produce a small amount of ATP, have a low rate of oxygen consumption, and also have a low mitochondrial mass and immature mitochondrial morphology [24,29,43]. In addition, glycolysis, producing lactate, was shown to be highly active in PSCs even in the presence of oxygen. These data were at first interpreted as evidence of the insignificant contribution of OXPHOS processes to the bioenergetics of PSCs. This interpretation correlated well with the hypothesis about low oxidative activity in PSCs due to the hypoxic conditions in their original niche that dominated in the first decade of the 2000s. However, in further studies, this concept has undergone significant adjustments. In 2011, it was shown that the ratio of the mitochondrial mass to the total protein and the ratio of the oxygen consumption rate to the mitochondrial mass are almost equivalent between normal human dermal fibroblasts and human PSCs (both ESCs and iPSCs) [29]. The data presented showed that, despite the morphological features of the mitochondrial network of PSCs and the active use of the glycolytic pathway for ATP synthesis, these cells possess functioning complexes of the mitochondrial respiratory chain and actively consume O_2_ [29]. Later, these observations were supported by metabolic flux analysis, performed by Turner et al. [26]. The results confirmed the high glycolytic nature of hESCs but, in addition, revealed a high activity of the TCA cycle in cellular mitochondria. The analysis showed that substrates used in this cycle by PSCs are mainly obtained not from glycolysis but using an alternative way—the process of catabolism of amino acids, in particular glutamine. In addition, the authors compared the metabolic characteristics of hESCs cultured at 2 and 21% O_2_ and found high metabolic plasticity of PSCs. They demonstrated that under different oxygen conditions, the activity of the OXPHOS pathways in PSCs can change, and at low oxygen content, hESCs can increase the activity of glycolysis to meet the total energy requirement of the cell. The results of further studies [44] confirmed that human PSCs (ESC and iPSC) rely both on glycolysis and glutamine oxidation for ATP generation, and hPSC viability critically depends on the presence of glutamine. Moreover, later it was found that metabolic shifts from glucose to glutamine oxidation and vice versa mediate the regulation of pluripotent cell identity. In-depth studies of both mouse and human PSCs have shown that pluripotency is not a discrete state but a wide spectrum of states, characterized by different potency and distinct metabolic profiles [45]. Terms ‘naïve’ and ‘primed’ pluripotency have been introduced, which allows to specify cells that are capable or incapable, respectively, of incorporation into a developing blastocyst generating chimeric embryos. Moreover, within the ‘naïve’ PSC population, a sub-population of cells with enhanced pluripotency (so-called ground state of ‘naïve’ pluripotency) was identified. Recently, Vardhana and colleagues [46] showed that transition towards this state increased the fraction of TCA cycle intermediates generated from glucose-derived carbons while decreasing the fraction of TCA cycle intermediates derived from glutamine.

Thus, the conducted studies have proven that for the production of ATP, PSCs rely on both oxygen-dependent and -independent metabolism to meet high requirements for energy production and substrates for anabolism needed for their high proliferative and functional activity. The idea of exclusively anaerobic metabolism with low mitochondrial activity has not been confirmed.

#### 2.2.2. Non-Mitochondrial ROS Production in PSCs

In cells of various types, non-mitochondrial ROS are produced by plenty of redox-active enzymes (xanthine oxidase, nitric oxide synthase, etc.). Between them, NADPH oxidases (NOXs) are considered to be the main and best-studied sources of non-mitochondrial ROS. NOXs is a group of transmembrane complexes consisting of NOX1-5 and DUOX1-2 that are able to transport electrons from NADPH to oxygen, generating either superoxide anion radical, which can be further transformed into H_2_O_2_ due to the activity of SOD enzymes, or directly H_2_O_2_. NOX1-3 complexes produce O_2_^−^· and interact with two membrane subunits (gp91-phox or its homologs, and p22-phox) that form the catalytic core of NOX, several cytosolic subunits (p47-phox, p67-phox, p40-phox), and the G-protein Rac, which are required for their assembly and activation [47]. Unlike NOX1-3, NOX4 generates H_2_O_2_ and interacts only with p22phox; therefore, it is considered a constitutively active isoform that is regulated at the level of transcript expression (reviewed in [48]). Since the superoxide anion dismutation rate is much higher than the reaction rate between superoxide and thiol groups of intracellular proteins, H_2_O_2_ is mainly considered to perform the signaling functions in the cell [49], being able to oxidize the cysteine residues of signal transduction proteins. Interestingly, simple NOX homologs were first discovered in early eukaryotes, in slime mold, and fungi [50]. A large-scale search for catalytic NOX subunits in unicellular and multicellular organisms showed the presence of these enzymes only in the latter, indicating the importance of NOX in ensuring the functioning of complex organisms and the coordination of intercellular signaling [51,52]. In mammals, NADPH oxidases can be found within the plasma membrane (NOX1-5 and DUOX1-2), mitochondrial membrane (NOX4), the endoplasmic reticulum (NOX2, NOX4, and NOX5), and nuclear membrane (NOX4 and NOX5). Due to the specific subcellular localization of different NADPH oxidases, ROS production is compartmentalized, leading to modulation of intracellular redox signal cascades [53]. The main function of NOXs is considered to be the regulation of multiple redox-dependent processes, such as proliferation, cell death, calcium signaling, cell differentiation, and reprogramming. NOX-produced ROS can increase intracellular calcium concentration by activating calcium channels, as well as directly oxidize some transcription factors such as NF-kB, HIF-1α, FOXOs, Nrf2, and p53 [54]. Most of these functions have been found for the first time in somatic cells; however, recently [55] it has been shown that NOX2 and NOX4 contribute to pluripotency maintenance and self-renewal of mouse iPSs (miPSCs) (see Section 2). Using the qPCR method, Kang and colleagues showed that miPSCs highly expressed *NOX2* and *NOX4*, while the mRNA levels of other NOX family members were much lower (*NOX1, DUOX1, DUOX2*) or undetectable (*NOX3* and *NOX5*). In addition, a number of other works [56,57,58] have shown that the expression of NADPH oxidases is temporarily increased during the differentiation of PSCs.

### 2.3. ROS Elimination in PSCs

All cells have a powerful system of antioxidant defense that allows precise control of intracellular ROS levels and a quick response to their threatening increases. Antioxidant defense system is an extensive group of both enzymatic and non-enzymatic substances. First-line defense enzymes are members of a superoxide dismutase (SOD) family. In addition to the already mentioned manganese SOD2 (localized in the mitochondrial matrix) and copper-zinc SOD1 (working in the cytoplasm, nucleus, and mitochondrial intermembrane space), cells also produce copper-zinc SOD3. The latter is secreted into the extracellular space and is anchored to the extracellular matrix and cell surface. SOD family provides dismutation of the superoxide produced by the mitochondrial and non-mitochondrial sources to H_2_O_2_. In turn, H_2_O_2_ scavenging is performed by several enzymatic systems. The first enzyme is catalase, although having a highly efficient reduction capacity for H_2_O_2_, it is localized mainly in peroxisomes and used for controlling the balance of H_2_O_2_ in these organelles [59]. One more potent H_2_O_2_ scavenger is glutathione peroxidase (GPX), a member of the cytosolic oxidoreductase family that inactivates H_2_O_2_ with the use of two molecules of glutathione (GSH), whose thiol groups donate their electrons to H_2_O_2_, forming a disulfide bond (GSSG). The reduction of oxidized glutathione occurs due to the activity of the glutathione reductase enzyme (GSR), which uses NADPH as a substrate [60]. Another highly efficient H_2_O_2_ detoxification enzyme is peroxiredoxin (PRX). Recent evidence suggests that PRX is not only capable of neutralizing H_2_O_2_, but it also enables relaying H_2_O_2_-derived oxidizing equivalents to other proteins, participating thus in the redox signaling events [61]. PRX, in turn, can be reduced by thioredoxins (TRX), a family of small proteins that are also actively involved in maintaining the redox homeostasis of a cell. In similarity to GSH, the presence of cysteine residues in the TRX structure allows the reduction of not only PRX but also many other intracellular proteins, as well as the regulation of signaling cascades by direct interaction with redox-active members of various signaling pathways [62].

PSCs, being parental cells assuring formation of all tissues of an organism, are equipped with an effective antioxidant system; however, the expression pattern of antioxidant genes differs from that revealed in the differentiated progenies of PSCs. Early studies of both murine and human ESCs and iPSCs have shown that expression of some antioxidant genes (*GPX2-4*, *SOD2*, *GSR)*, as well as accumulation of some antioxidant proteins (SOD2, catalase), is markedly reduced during PSC differentiation [28,35,36,38]. Basing on these observations, it was suggested that pluripotent cells possess a highly potent antioxidant system, more effective than that of their differentiated descendants. However, several studies, aimed to compare the transcriptome profile of PSCs and their differentiated progenies or various cells from adult tissues, did not reveal antioxidant enzyme genes in the lists of differentially expressed genes [63,64]. Similarly, genetic screenings or transcriptome profiling did not find principal antioxidant enzymes in the sets of genes that were demonstrated to be crucial for initiation and maintenance of pluripotency [65,66,67]. In addition, functional tests for the PSC resistance to oxidative stress did not confirm the hypothesis about the enhanced antioxidant protection of these cells. The comparison of the rate constants of the H_2_O_2_ elimination between hESCs, their differentiated offspring, and adult mesenchymal stem cells showed that hESCs eliminate exogenous H_2_O_2_ even slower than any of the two differentiated cells listed above. However, when normalizing this constant not per cell, but per cell protein, the values became equal for hESCs and their differentiated progenies, indicating the same functional activity of their antioxidant systems [30]. Like many other redox parameters of PSCs (such as low ROS level, oxygen consumption, mitochondrial mass), the poor ability of these cells to eliminate exogenous H_2_O_2_ appears to be related to their small size, which results in fewer H_2_O_2_ scavengers per cell in comparison to PSC differentiated counterparts.

### 2.4. Oxidative Stress Response in PSCs

The levels of ROS that exceed the physiological norm (‘oxidative distress’) are known to cause a strong damaging effect on cells. The fate of cells that survived oxidative stress depends on many factors: the potency of antioxidative defense, the ability of a cell to repair the damage, signaling pathways that shape stress response programs, etc. For example, in response to genotoxic impacts (including oxidative stress), transformed cells usually initiate apoptosis programs, while many normal cells of an adult organism, such as fibroblasts and mesenchymal stem cells, are prone to activating premature senescence [68,69]. Predisposition to the certain stress response program determines the viability of cells after damaging factor withdrawal.

When studying the activity of DNA damage repair response in murine PSCs, Saretzki et al. found that radiation-induced DNA strand break repair is superior in mESCs in comparison to differentiated mESC progenies or mouse embryonic fibroblasts [28]. mESCs repaired DNA faster than 3T3 mouse fibroblasts and, when cultured under hyperoxia (40% O_2_), only slightly reduce their proliferation rate, while mouse embryonic fibroblasts stopped dividing. It was found also that many genes involved in stress response (heat shock genes, *BMI1*, *ERCC4*) decreased their expression during mESC differentiation [28]. Another study [70] also showed that, in comparison to somatic cells, both human ESCs and iPSCs possess high levels of DNA repair proteins (RAD51, Ku70, XLF, DNA LigIIIα, XRCC1, and PARP1) that participate in the double-strand break, single-strand break, and base excision repair pathways. Using functional tests (plasmid-based repair assay), Fan et al. showed that both hESCs and human iPSCs (hiPSCs) demonstrated elevated efficacy of nonhomologous end-joining, one of the main pathways for DNA double-strand breaks repair [70].

However, when assessing the cytotoxic effect of exogenous H_2_O_2_ on hESCs and their differentiated offspring, the cytotoxic dose (estimated as the number of H_2_O_2_ moles per one cell) turned out to be ten times lower in hESCs, and when normalized to a cellular protein, which allows taking into account the difference in the cell volume—two times lower [30]. Thus, despite the efficient system of DNA damage repair in PSCs, their resistance to the cytotoxic effect of oxidative stress turned out to be low. One of the reasons for this phenomenon was found in studies that showed both mESCs and hESCs quickly and efficiently eliminate oxidatively damaged cells by apoptosis, while their differentiated counterparts do not die, but undergo premature senescence [71,72].

Thus, even though many parameters of redox homeostasis (overall ROS level and mitochondrial mass normalized per cell protein, oxygen consumption rate normalized per mitochondrial mass, activity of redox metabolism pathways, potency of the antioxidant system) turned out to be similar in PSCs and their differentiated progenies, the response of pluripotent cells to oxidative stress is fundamentally different from that of differentiated cells. PSCs turned out to be highly sensitive to the oxidative load due to their small size, but simultaneously they are more protected from the damaging effects of stress due to their ability to effectively repair DNA breaks and eliminate damage at the level of cell population.

## 3. Redox Signaling in Pluripotent Stem Cells

A lot of proteins participating in intracellular signaling cascades have thiol-rich cysteine residues in their structure, including in active centers. During oxidation, thiol groups lose hydrogen atoms and form disulfide bonds (or “disulfide bridges”) between their sulfur atoms that lead to a change in the conformation of the signaling protein and ensures signal transduction [49]. It is important to note that the formation of disulfide bonds is a reversible process. The reduction of thiol groups occurs due to the activity of thiol–disulfide exchange enzymes, which mainly belong to glutathione- and thioredoxin-dependent enzymatic systems. Thus, the mechanism of redox signaling is a kind of switching between active/inactive protein states where ROS play the role of “fingers” pressing the switches. Different subcellular localization of sources and short lifetimes of ROS lead to compartmentalization and spatiotemporal distribution of intracellular redox signals, which results in the timely activation of strictly defined signaling pathways.

PSCs perform two main functions: (1) they actively proliferate to maintain their population, and (2) they are able to differentiate into various cell types of the three germ layers on cue. A number of studies confirm the involvement of ROS in the regulation of the self-renewal process and differentiation of PSCs, as well as induction of pluripotency.

### 3.1. ROS and Proliferation in PSCs

To date, the involvement of ROS in the redox regulation of PSC proliferation has been proven for both murine and human ESCs and iPSCs [55,73,74,75,76]. Evidence for this involvement is based on a variety of observations. First, PSC proliferation can be promoted by overexpression of complexes that directly produce ROS, such as NOX2 and NOX4, as well as by the addition of small doses of exogenous H_2_O_2_ [55,74]. Further, stimulation of signaling cascades leading to an increase in intracellular ROS (such as stimulation of the peroxisome proliferator-activated receptor PPARδ) also has a mitogenic effect on PSCs [73]. At the same time, a decrease in the ROS level upon inhibition of NOX family enzymes using small interfering RNAs, or their widespread inhibitors (DPI, Apocynin), leads to a slowdown in proliferation and a decrease in the number of PSC colonies [55,76].

**Figure 2:**
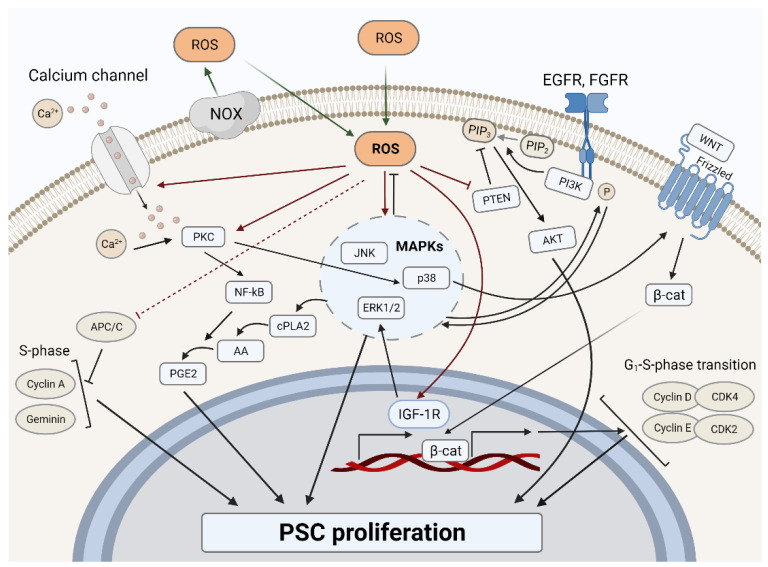
Overview of ROS-dependent signaling cascades involved in PSC proliferation regulation. ROS, reactive oxygen species; NOX, NADPH oxidase; FGFR, fibroblast growth factor receptor; EGFR, epidermal growth factor receptor; PKC, protein kinase C; NF-kB, nuclear factor kappa-light-chain-enhancer of activated B cells; cPLA2, phospholipase A2; AA, arachidonic acid; PGE2, prostaglandin E2; APC/C, anaphase-promoting complex/cyclosome; MAPK, mitogen-activated protein kinase; ERK, extracellular signal-related kinases; JNK, c-Jun N-terminal kinases; p38, p38 kinase; AKT, protein kinase B; MAPK, mitogen-activated protein kinase; PTEN, phosphatase and tensin homolog deleted on chromosome 10; PI3K, phosphatydylinositol-4,5-bisphosphate 3-kinase; PIP3, phosphatidylinositol 3,4,5-triphosphate; PIP2, phosphatidylinositol 4,5-bisphosphate; β-cat, β-catenin; IGF-1R, insulin-like growth factor 1 receptor; CDK, cyclin-dependent kinase; PSC, pluripotent stem cell.

Similar to somatic (both stem and non-stem) cells [77,78,79,80,81,82,83], ROS are involved in the regulation of PSC proliferation at two levels: (1) via ROS-dependent mitogenic signal transduction from cytoplasmic membrane receptors or intracellular receptors; (2) by direct regulation of the level or activity of proteins participating in the cell cycle (Figure 2).

#### 3.1.1. ROS-Dependent Mitogenic Stimulation

The main pathways responsible for mitogenic stimulation of cells are mitogen-activated protein kinases (MAPK) and phosphoinositide 3-kinase/protein kinase B (PI3K/Akt) signaling pathways. Serine-threonine MAPK cascades are organized as modular pathways in which the activation of upstream MAP kinase kinase kinase (MAPKKK or MAP3K) through the binding of a specific ligand to the receptor leads to the activation of downstream MAP kinase kinase (MAPKK), which further activate terminal MAP kinase [84]. In according to terminal kinases, MAPK signaling pathways are subdivided into three subgroups: extracellular signal-regulated kinases (ERK1/2), N-terminal c-Jun kinases (JNK), and p38 MAPK. Typically, the ERK pathway is considered to be activated by growth and survival factors, while the JNK and p38 MAPK pathways are activated in response to stressful stimuli [85]. Despite the fact that each MAPK module is activated in response to the stimulation of specific receptors by specific ligands, activated downstream elements of different modules can also interact with each other, leading to ligand-independent, mutually mediated activation of signaling pathways. Once activated, MAPKs can phosphorylate many intracellular targets, including transcription factors, nuclear pore proteins, membrane transporters, cytoskeletal elements, etc. [86]. MAPK phosphatases (MKP) are negative regulators of this cascade, which provide modulation of the duration, magnitude, and spatiotemporal profile of MAPK activity in response to both physiological and pathological stimuli [87]. The involvement of ROS in the regulation of the activity of mitogenic cascades has been shown for many somatic stem cells [48,77,78,79], and membrane enzymes of the NOX family were identified as the main sources of signaling ROS for these pathways. One of the ROS targets were found to be upstream MAP3Ks, among which ASK1 (apoptosis signal-regulating kinase 1) has been extensively characterized as a ROS-responsive kinase. When being inactive, ASK1 is bound to reduced TRX protein. Upon oxidation, the disulfide bond forms between the cysteine residues in the active site of TRX that makes the protein dissociate from the kinase, thereby restoring its activity [88]. Moreover, ROS was shown to be involved in the activation of MAPK cascades through transactivation of the growth factor receptors in a ligand-independent way, interacting directly with their thiol groups [89]. Finally, ROS have been shown to play a significant role in regulating MAPK activity by oxidative inactivation of MKP [54,90].

The PI3K/Akt pathway is important for many critical cellular functions, including protein synthesis, cell cycle progression, proliferation, apoptosis, autophagy, response to growth factors, certain hormones, and cytokines. Activated PI3K catalyzes the synthesis of phosphatidylinositol-3,4,5-triphosphate (PIP3) from phosphatidylinositol-4,5-bisphosphate (PIP2) [79], and PIP3, in turn, activates protein kinase B (AKT). For somatic cells, it was shown that ROS can directly activate PI3K as well as inactivate the inhibitor of PIP3 synthesis, phosphatase, and tensin homolog (PTEN) [91]. In addition, similar to the situation with MAPK kinases, ROS can deactivate the AKT-inhibiting phosphatase 2A (PP2A), thereby activating the downstream signaling pathway.

The main redox-dependent mitogenic pathways identified in PSCs are p38, JNK, and ERK1/2 kinases, the phosphorylation of which decreases upon inhibition of NOXs and increases together with the elevation of intracellular ROS due to PPAR activation [92]. Activation of MAPK cascades in PSCs can occur directly, through an increase in the mRNA level of growth factor receptors [55], as well as indirectly, for example, by activation of protein kinase C (PKC) caused by an increase in the concentration of intracellular calcium due to H_2_O_2_-induced oxidation of calcium channel proteins [92]. H_2_O_2_-mediated activation of MAPKs through PKC led to increased arachidonic acid release, NF-kB activity, and prostaglandin E2 production, consequently stimulating proliferation in mESCs. In addition, it has been shown that ROS are also involved in the activation of the PI3K/Akt signaling pathway in PSCs [93]. It was found that ROS can stimulate PI3K-mediated conversion of PIP2 to PIP3 by inhibiting the PI3K antagonist PTEN. Besides, it was also demonstrated that both in the case of the MAPK cascades and the PI3K/Akt pathway, ROS can stimulate the activation of upstream elements of these pathways, in particular, enhancing the phosphorylation of the growth factor receptors, both in the presence and in the absence of an activating ligand [74,93].

#### 3.1.2. ROS-Dependent Cell Cycle Regulation

The main regulators of the cell cycle are known to be cyclin-dependent kinases (CDK), which work in a complex with cyclin proteins, and each phase of the cycle is associated with the activity of the certain protein complex [94]. In somatic cells, the activity of CDK’s, as well as the level of cyclins’ expression, change throughout the cell cycle, thereby providing a transition between its phases. G_1_ phase is regulated by the complex CDK4/CDK6 (CDK4/6) and cyclin D. Cyclin D, in turn, is expressed in response to extracellular proliferative signals. CDK4/6, in association with cyclin D, initiates phosphorylation of the retinoblastoma protein (pRb). In the phosphorylated state, pRb is detached from the transcription factors of the E2F family, which allows them to activate the transcription of the important cell cycle regulators—cyclin E, cyclin A, phosphatase Cdc25—thus providing the adequate progression of the next S-phase of the cell cycle. The CDK2/cyclin E complex enhances pRb phosphorylation, creating a positive feedback loop. The release of E2F factors contributes to the initiation of DNA synthesis. During the synthesis phase, the CDK2/cyclin A complex works. At the beginning of the G2/M phase, cyclin A binds to CDK1, stimulating the synthesis of cyclin B, which then binds to CDK1 instead of cyclin A, and provides the beginning of cell division prophase. The modulation of the cyclin levels, as well as of a number of important cell cycle regulator proteins, is provided by their timely proteasome degradation with the participation of the anaphase-promoting complex/cyclosome ubiquitin ligase (APC/C) [95]. APC/C, when interacting with its coactivators, is active from prophase to the late G_1_ phase of the cycle. This complex provides an adequate progression of mitotic division, as well as mitotic exit, by causing the degradation of mitotic cyclins. For the transition to the S phase and further progression of the cycle, the APC/C activity is suppressed by the early mitotic inhibitor-1 (Emi1) protein, which is under the control of E2F transcription factors. Emi1 prevents coactivators from assembling with APC by blocking its active center. Inactivation of APC/C leads to the accumulation of cyclin A and other regulators of the synthetic phase, such as geminin, which play a crucial role in prevention of re-replication of DNA.

The participation of ROS in the regulation of the cell cycle of somatic cells has been proven by a number of studies. It was found that the ROS level oscillates in accordance with the cell cycle, and the peak of this oscillation occurs in the early S-phase [80,96,97]. Following these observations, a decrease in the ROS level by using various antioxidants leads to a block of the transition from the G_1_ to the S-phase of the cell cycle [80,98]. Antioxidant-induced cycle blocking is characterized by active CDK4/6-cyclin D and CDK2-cyclin E kinases, inactive hyperphosphorylated pRb, but simultaneously—by the inability to accumulate cyclin A. The continuing activity of APC/C complex, targeting cyclin A for degradation, was found to be the main cause for the G_1_-S arrest of cells treated with antioxidants. An APC/C inhibitor, Emi1, which was also destabilized with a decrease in the ROS level, was proposed as the main candidate for the role of a redox regulator of this process. Emi1 is a zinc-binding protein that contains many cysteine residues and, therefore, could be redox-regulated [99].

Unlike somatic cells, PSC cultures are characterized by a short doubling time (15–16 h), shortened G_1_ and G_2_ phases, and the absence of a G_0_ phase. Initially, the cell cycle of PSCs was studied using the mESC model. mESCs exhibit constitutive activity of CDK and other regulators throughout the cycle, inactivation of the restriction point in the G_1_ phase due to the maintenance of a constantly hyperphosphorylated (inactive) pRb, and almost complete absence of cyclins D expression [100]. In comparison to mESCs, most of the cell cycle regulators in hESCs oscillate, depending on the cell cycle phase, the G_1_ restriction point is active, and the APC/C ubiquitin ligase complex is deactivated from the late G_1_ to the mitosis phase [101,102,103]. When compared with somatic cells, the observed oscillations of cell cycle proteins have a lower amplitude due to the high expression of the negative regulators of these oscillations, including the APC/C inhibitor of the Emi1 protein.

The history of the study of redox-dependent events within the embryonic cycle dates back to long before the discovery of PSCs, to the 1930s of the last century, when L. Rapkine first revealed the modulation of the level of non-protein thiols (i.e., GSH) in different phases of the cell cycle of sea urchin eggs [104]. A more detailed study carried out on the same model showed that the number of disulfide groups increased towards the prophase of mitosis and then decreased during anaphase [105]. Recent studies carried out on the Xenopus embryos model using the highly sensitive genetically encoded H_2_O_2_ sensor HyPer confirmed these observations and showed that fertilization triggers a rapid increase in ROS levels, which oscillate with each cell division [106]. Inhibition of NOXs by commonly used agents such as apocynin and DPI did not change the observed H_2_O_2_ increase, while blocking the mitochondrial complexes, which are involved in ROS production, led to suppression of oscillations.

Turning to a review of the data obtained directly for PSCs, it should be noted that the redox regulation of proteins that control the progression of the pluripotent cycle has been little investigated. Recent studies by our group [107], carried out using hESC and hiPSCs, showed that similarly to the cells of Xenopus embryos and sea urchin eggs, as well as human somatic cells, the ROS level oscillates with each hPSC division, being maximal in the synthetic phase of the cell cycle. In accordance with these observations, targeted lowering of ROS levels using antioxidants or a NOX inhibitor DPI slows the initiation and progression of the S-phase. Analyzing possible molecular targets of ROS in the PSC cycle, we found that the cell cycle hindering effect induced in PSC by antioxidants is accompanied, similarly to somatic cells, by a drop in the level of the main synthetic phase regulators—cyclin A and geminin. Accordingly, since it is known that cyclin A and geminin are among the main substrates for APC/C ubiquitin ligase, it can be assumed that redox mechanisms of proliferation regulation, which are similar to somatic cells, may exist in PSCs, arising from the direct or indirect dependence of APC/C activity on the ROS level. However, this hypothesis certainly requires additional studies.

### 3.2. ROS and Differentiation of PSCs

In most cases, PSC differentiation process starts with the formation of embryoid bodies (EBs)—three-dimensional aggregates that can be assembled from PSCs using various methods, and the most common of them is the EB self-assembling due to the cultivation in non-adhesive culture dishes [108]. These aggregates mimic the developing embryo, allowing PSCs to differentiate into various cell lines under the influence of certain stimuli. Analysis of the ROS level modulation and the level of various redox enzymes in cells at different time points after EB formation performed in a number of studies resulted in identifying the redox-dependent phases and signaling pathways inherent to PSC differentiation process. As a rule, short-term activation of redox-active systems was observed at the early stage of PSC differentiation [109,110], preceding the activation of expression of differentiation-specific genes. In particular, Sauer and colleagues [110] revealed a temporal increase in the intracellular ROS level in EBs with a maximum found on days 2–3 after induction of cardiogenic differentiation. Yanes and colleagues [109] also showed a pro-oxidative change in the redox status of mESCs at the early stage of differentiation into cardiomyocytes and neurons by carrying out comprehensive metabolic profiling of differentiating cells. According to these data, the GSH/GSSH ratio (inversely correlating with oxidative load on the cell) gradually decreased, while the level of ascorbic acid (important intracellular reducing agent) steeply increased by day 4 of cultivation in EB, thus indicating the coordinated work of cell redox systems aimed at ensuring the adequate signaling function of ROS at the early stage of differentiation. It is important to note, that both abovementioned studies [109,110] revealed that, after the initial variations, ROS level and GSH/GSSH ratio in differentiating cells returned to their initial values by days 11 and 7 respectively, providing the restoration of the basal redox state. At the same time, evidence exists that if the ROS level remains high at later stages of differentiation process; this, on the contrary, leads to a decrease in the efficiency of differentiation [111].

To track overall redox-dependent metabolic changes during PSC differentiation, the relative abundance of metabolites in ESCs and their differentiated progenies (neurons and cardiomyocytes) has been quantified [109]. The metabolomic approach identified a specific ‘metabolic signature’ of undifferentiated mESCs, characterized by the presence of a large number of unsaturated metabolites enriched with carbon–carbon double bonds. As chemical unsaturations such as carbon–carbon double bonds are highly reactive under oxidative conditions, the abundance of these species in mESCs was hypothesized to be important in mediating differentiation through regulation of cellular redox status. In support of this hypothesis, the enrichment of undifferentiated mESCs with the metabolites produced by and involved in oxidation metabolism led to the stimulation of cardiogenic and neurogenic differentiation [109].

Interestingly, while all of the abovementioned studies pointed out the essentiality of early pro-oxidative events for stimulation of differentiation process in PSCs, an increase in the ROS level was also recently found to stimulate PSC dedifferentiation program. It is known that a small percentage (less than 1%) of totipotent-like or 2C-like cells, which are able to differentiate into both embryonic and extraembryonic cells, can arise spontaneously in ESC cultures [112]. Zhang and colleagues [113] proved that intracellular ROS levels increase, stimulated by cell treatments either with exogenous H_2_O_2_ or ROS-inducing small molecules, promoting the activation of totipotent-like state in mESCs.

A number of studies showed that the ROS level increase observed at the early stages of PSC differentiation was associated with the stimulated activation of NOXs. For example, Sauer et al. [110] found that the growing ROS level in EBs correlated with an increase in the expression of p67phos subunit of NADPH oxidase. Further, Buggisch and colleagues demonstrated a sequential increase in the expression of NOX1, NOX2, NOX4 genes in EBs during cardiogenic differentiation [56]. Furthermore, the application of NOX inhibitors, such as DPI, led to the suppression of observed ROS increase [110]. Different types of NOXs were found to contribute to the stimulation of various differentiation directions. So, NOX4-generated ROS were shown to mediate cardiotypic and smooth muscle differentiation of ESCs [57,58]. NOX2 activity was associated with the formation of phagocytic cells [114]. NOX1 together with NOX4 participated in endothelium generation [115]. In addition, NOX-produced H_2_O_2_ was found to be a transducer of cardiovascular differentiation of PSCs, caused by mechanical stress [116] or a constant electric field [114,117,118]. In addition to NOX-generated ROS, a contribution of mitochondrial ROS to the early pro-oxidative effects has also been proved [119]. Increased release of mitoROS to the cytoplasm was stimulated by the temporary opening of the mitochondrial pore (mPTP) [111,120,121,122].

By now, redox regulation of PSC differentiation is thought to be mainly associated with MAPK and PI3K signaling [117,123]. According to Sauer and colleagues [110], cytokine-PI3K-NOXs cascade generates an initial signal resulting in the ROS upregulation during cardiac differentiation of mESCs. As for MAPK cascades, all the main members of the MAPK family (JNK, ERK1/2, and p38 kinase) were found to be involved in the redox-regulated PSC differentiation. JNK was shown to be activated by NOX4-produced H_2_O_2_, subsequently ensuring the upregulation of GATA-4 transcription, one of the earliest markers of cardiotypic differentiation [124]. At the same time, an excessive level of H_2_O_2_ leads to the ubiquitination and degradation of this protein and disruption of the differentiation process [125]. According to [126], an increase in the ROS level in mESCs induced by the knockout of PRX1,2 promoted the initiation of early stages of neurogenesis due to the activation of JNK and ERK1/2. In addition, transient phosphorylation of the abovementioned MAPKs, mediated by an increase in endogenous ROS caused by the administration of glutathione inhibitor buthionine sulfoximine (BSO), stimulated differentiation of hESCs in the bipotent meso-endodermal direction [127]. Several studies on mESCs demonstrated that NOX4-produced ROS activated p38 MAPK, which promoted nuclear translocation of myocyte enhancer factor 2C (MEF2C), another early marker of cardiogenic differentiation [57,128]. Moreover, the addition of low concentrations of H_2_O_2_ and menadione during mESC cardiac differentiation led to an increase in the number of “beating” EBs (with observed contractions), as well as an increase in the expression of cardio-specific genes *MEF2C* and *GATA-4* [56,110]. In support of these observations, inhibition of NOX activity in these experiments led to strictly opposite effects [56,110]. Interestingly, although the experiments on hESCs [127] revealed redox stimulation of JNK and ERK1/2 signaling, an increase in the intracellular ROS level, on the contrary, led to the inactivation of the p38 MAPK signaling cascade. This may evidence of some specific features of redox-dependent differentiation signaling associated with different types of cells and/or different stages of the differentiation process [111].

### 3.3. ROS and Induction of Pluripotency

Development of a technology for reprogramming somatic cells into a pluripotent state and the creation of induced pluripotent stem cells (iPSCs) have become a breakthrough in both basic science and applied biology at the beginning of the 2000s [20,21]. This approach avoids ethical problems and also allows obtaining PSCs that are compatible with the immune system of a certain individual. In addition, this method offers a unique experimental system for investigating key issues related to the regulation of pluripotency, determination of cell fate, and epigenetic regulation. Reprogramming is considered a multistep process mediated by various transcription factors, which includes several phases—initiation, maturation, and stabilization [129]. During the initiation phase, a major transcriptional shift occurs, which leads to the sequential activation of the pluripotent gene expression in the maturation phase, which ends in the stabilization phase, where cells begin to self-renew independently of the introduced transgenic sequence. At the moment, the main disadvantage of this technology is the duration and very low efficiency of this process, as a very small number of cells entering the initiation phase of reprogramming move to the next stage of the process. According to the data of genome-wide analysis of gene expression, protein levels, as well as metabolomic analysis, a cell begins large-scale metabolic shift almost immediately after the initiation of the reprogramming process, and one of the most important rearrangements is the metabolic shift from oxidative phosphorylation to the predominant glycolytic pathway of energy production inherent in PSCs [130,131,132,133]. However, before making the transition, a cell undergoes a state of temporary hyper-energetic metabolism, which combines both the high activity of oxidative phosphorylation and glycolysis [134,135,136]. This state is provided by an “explosion” of OXPHOS activity, accompanied by an increase in the amount of ETC protein complexes II, III, and V and observed already on the 3rd day of reprogramming [131,137]. In addition, similar to the early stages of the differentiation process, transient activation of mPTP is involved in the early phase of somatic cell reprogramming. Short-term mPTP opening triggers a mitochondrial ROS/miR-101c pathway that enhances plant homeodomain finger protein 8 (PHF8)-mediated H3K9me2/H3K27me3 demethylation of pluripotency genes [138]. Accordingly, ROS level temporally increases at the earliest stages of reprogramming with a peak around days 4–8, depending on the type of reprogramming system [139,140,141,142]. According to Zhou and colleagues, the enhanced generation of ROS correlates with NOX2 upregulation, and the use of antioxidants, such as EUK-134 and ebselen, as well as specific NOX inhibitors (DPI and apocynin), lead to a decrease in the efficiency of reprogramming. In addition, mito-targeted antioxidant (mito-Tempo) also causes, albeit not so dramatic, a decrease in the efficiency of reprogramming, indicating that ROS produced by mitochondria also contribute to the redox regulation of this process. Another important result of this work is the fact that the use of antioxidants and NOX inhibitors negatively affects the reprogramming only when used at the earliest stages of this process (up to 7 days), while their use at later stages does not affect dedifferentiation. This observation indicates the importance of the ROS level increase and redox regulation precisely at the early stages of the pluripotency induction process. Moreover, according to [141], the supplementation of reprogramming cells with the antioxidants (N-acetyl-L-cysteine and vitamin C) during reprogramming did not affect the efficiency but reduced the level of genomic aberrations arising during reprogramming. At the same time, Esteban and colleagues demonstrated that vitamin C (ascorbic acid), but not other antioxidants (such as N-acetyl-L-cysteine, resveratrol, reduced glutathione, vitamin B), increased the efficiency of reprogramming, suggesting that the activity of vitamin C may be independent of its antioxidant properties [142].

**Figure 3:**
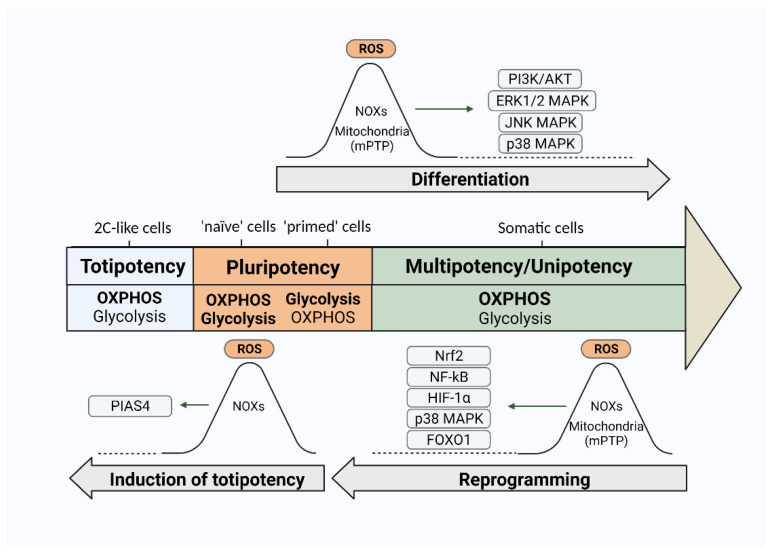
Redox signaling pathways participating in the regulation of cell differentiation, induction of pluri- and totipotency. The onset of these programs is accompanied by the early short-term ROS level increase, which mediates/stimulates the rearrangements of cell phenotypes and large-scale metabolic shifts. These shifts determine the prevalent energy production pathway corresponding to each cell phenotype. ROS, reactive oxygen species; NOX, NADPH oxidase; MP, mitochondrial pore, OXPHOS, oxidative phosphorylation pathway; PI3K, phosphatydylinositol-4,5-bisphosphate 3-kinase; AKT, protein kinase B; MAPK, mitogen-activated protein kinase; ERK, extracellular signal-related kinases; JNK, c-Jun N-terminal kinases; p38, p38 kinase; MAPK, mitogen-activated protein kinase; Nrf2, nuclear factor erythroid 2-related factor 2; NF-kB, nuclear factor kappa-light-chain-enhancer of activated B cells; HIF-1α, hypoxia-inducible factor 1α; FOXO1, forkhead box protein O1; PIAS4, protein inhibitor of activated STAT 4, 2C-like cells, transient cell population within PSC cell cultures that expresses high levels of transcripts found in two-cell (2C) embryos.

According to the latest data [140,143], the bioenergetic shift during cell reprogramming is provided by the activation of several ROS-dependent transcription factors, such as NF-kB, Nrf2, and HIF-1α. Hawkins and colleagues demonstrated ROS-associated activation of Nrf2 on the 8th day of reprogramming, followed by a wave of HIF1-α activation (on the 11th day), which subsequently induces an increase in the expression of genes related to the glycolytic pathway of energy production. According to this study, from days 8 to 14, actively proliferating cells use mainly OXPHOS, while glucose is redirected to PPP for increased nucleotide synthesis, thus providing proliferation capacity. An increase in the level of ROS activates Nrf2, which, in turn, triggers HIF1-α that ensures the final bioenergetic shift. Thus, both for PSC differentiation and dedifferentiation and induction of pluripotency, an early increase in the ROS level is of fundamental importance, triggering various signaling cascades that stimulate large-scale metabolic shifts (Figure 3).

In addition to redox regulation via the ROS-dependent transcription factors, several other redox-sensitive signaling pathways are implicated in cell reprogramming. Stress-activated p38 MAPK signaling cascade has been shown to play a critical role in the progression through all phases of reprogramming [144]. Inhibition of these cascades leads to a decrease in the activation of the pluripotent genes’ expression, disruption of MET transition, and an increase in the expression of differentiation markers, which ultimately leads to a decrease in the efficiency of the reprogramming process and a complete loss of iPSCs at the stage of stabilization [144]. The transcription factor FOXO1 is also essential for the acquiring and maintenance of PSC pluripotency, which is probably mediated through the direct control by FOXO1 of *OCT4* and *SOX2* gene expression via occupation and activation of their respective promoters [145]. This transcription factor was also identified as a mediator of the process of cellular pluripotency induction—the peak of its activation preceded the start of the *OCT4* gene expression, and knockdown led to a decrease in the number of formed iPSC colonies [146]. The activity of both p38α MAPK and FOXO1 signaling pathways is known to be dependent on the level of intracellular ROS.

## 4. Concluding Remarks

The significance of research on physiology of PSCs, both ESCs, and iPSCs, can hardly be overestimated. Pluripotency is a continuum of cellular states that includes not only ‘naïve’ to ‘primed’ lineage but, in fact, the wide range of metabolically different states, which are shaped, among other things, by cultivation under different oxygen conditions (hypoxia and normoxia). Highly active glycolysis in PSCs is usually identified with the suppressed OXPHOS pathway, and the rationale of this metabolic rearrangement is commonly explained by the urge to decrease the amount of “harmful” ROS to protect intracellular macromolecules from oxidative damage. However, recent studies show that, in each pluripotent state, the activity of OXPHOS fed by the TCA cycle, which in turn can be fueled either by glycolysis or other carbon sources such as glutamine, has been revealed. Accordingly, we assume that pluripotency should not be unequivocally aligned with any specific redox status and, even more so, with a decreased level of ROS. Conversely, the generalization of numerous data demonstrates that under normoxic conditions, key redox parameters of cultivated PSCs (ROS level, mitochondrial mass, rates of oxygen consumption, and ROS removal), when calculated on a per cell protein basis, have much in common with that of their differentiated progeny cells. Thus, pluripotency is not associated with exclusively anaerobic metabolism but is supported by the dynamic balance between glycolysis and OXPHOS energy pathways. Comparing the redox profile of PSCs and somatic cells, we suggest not to consider PSCs as unique cells but as cells in which the features of all adult cells are laid a priori. After differentiation, the adult cells can either exploit mitochondrial ETC or glycolysis (ex: erythrocytes) as the main path of energy production.

In addition, numerous studies reveal that, similar to somatic cells, ROS are involved in the regulation of the most important physiological functions of PSCs—proliferation and differentiation. Interestingly, the mechanisms of ROS-dependent regulation of PSC proliferation resemble that of somatic cells. ROS are involved both in mitogenic cascades activation and in pluripotent cell cycle regulation, and progression through the cell cycle phases is accompanied by the ROS level modulation. It is worth noting that cell cycle-coupled ROS oscillations were observed not only for human PSCs but also in the embryonic cycle of other organisms (sea urchin and Xenopus), evidencing conservative redox processes that govern cell division. Along with cell proliferation, the processes of cell differentiation, induction of pluri- and even totipotency also appear to be regulated by ROS. The onset of these programs is accompanied by the early short-term ROS level increase that, in turn, mediate large-scale metabolic shifts providing the rearrangements of cell phenotypes.

In summary, we came to the conclusion that PSCs possess metabolic plasticity and are able to adapt to both hypoxia and normoxia conditions while maintaining pluripotency. Moreover, PSCs preserve redox homeostasis and redox signaling characteristics similar to that in somatic cells. While the in-depth studies in the future may possibly shed light on some specific features of the redox processes in various types of cells, our current knowledge allows us to draw only general conclusions about highly conservative principles of aerobic life that unite both PSCs and differentiated cells.
